# Investigating adverse effects of chronic dietary exposure to herbicide glyphosate on zootechnical characteristics and clinical, biochemical and immunological blood parameters in broiler chickens

**DOI:** 10.1007/s11259-023-10195-x

**Published:** 2023-08-18

**Authors:** Elena A. Yildirim, Georgi Yu. Laptev, Daria G. Tiurina, Elena P. Gorfunkel, Larisa A. Ilina, Valentina A. Filippova, Andrei V. Dubrovin, Evgeni A. Brazhnik, Natalia I. Novikova, Veronika Kh. Melikidi, Kseniya A. Kalitkina, Ekaterina S. Ponomareva, Darren K. Griffin, Michael N. Romanov

**Affiliations:** 1 BIOTROF+ Ltd, Pushkin, St. Petersburg, Russia; 2 Federal State Budgetary Educational Institution of Higher Education “St. Petersburg State Agrarian University”, Pushkin, St. Petersburg, Russia; 3 School of Biosciences, University of Kent, Canterbury, UK; 4 L. K. Ernst Federal Research Centre for Animal Husbandry, Dubrovitsy, Podolsk, Moscow Oblast Russia

**Keywords:** Glyphosate, Broilers, Immunity, Productivity, Biochemical parameters of blood serum

## Abstract

Although the herbicide glyphosate is widely used globally and considered safe, more evidence of its adverse effects on animals and humans is accumulating. The present investigation was aimed at evaluating the impact of different glyphosate concentrations on zootechnical characteristics and clinical, biochemical and immunological blood parameters in Ross 308 broiler chickens. Four groups were employed, including untreated control and three experimental groups fed diets enriched with glyphosate at doses of 10, 20 and 100 ppm that conformed to 0.5, 1 and 5 maximum residue limits, respectively. The results showed that glyphosate is a stress factor triggering a multifaceted effect on important blood parameters (e.g., white blood cell and phagocytic counts), which was shown for the first time in the experiments involving productive meat-type poultry. It was first revealed that glyphosate-induced changes in blood parameters may be related to a negative impact on the zootechnical characteristics including the digestive tract organ development and body weight gain. The study findings suggested that exposure to glyphosate in the feedstuffs can adversely affect the physiological condition and productivity of broilers.

**Supplementary Information:**

The online version contains supplementary material available at 10.1007/s11259-023-10195-x.

## Introduction

Glyphosate (GLY), or *N*-(phosphonomethyl)glycine, is the most commonly used nonselective herbicide worldwide (Szekacs and Darvas 2018; Heymann et al. 2021). It was marketed under the Roundup® brand name starting in 1974, and after the patent expired in 1991, several companies gained approval and commercialized their own formulations under various trade names like Bronco, Glifonox, KleenUp, etc. (Szekacs and Darvas 2018; Vicini et al. 2019, 2021; Foldager et al. 2021). Glyphosate is used to kill weeds during the introduction of abandoned and fallow lands into circulation, in the care of fallows, in the pre-sowing cultivation of fields and in the minimum cultivation technologies of many crops (Vicini et al. 2019). The estimated amount of GLY entering the food chain through genetically modified, GLY-resistant crops (mainly soybeans) is several thousand tons per year (Bohn and Millstone 2019). The poultry industry is the largest consumer of soybean meal, accounting for about half of all soybean meal produced, which suggests dietary exposures to GLY residues in the feedstuffs for birds and in the poultry products consumed by humans of (Vicini et al. 2021).

GLY has been considered a safe chemical, with 99% of GLY residues in food being below the European maximum residue limits (MRLs) or U.S. Environmental Protection Agency tolerances (Hammond et al. 1996; Sidhu et al. 2000; Vicini et al. 2019, 2021). There is a limited evidence for impact of GLY on liver histology and biochemical blood parameters in animals (e.g., Heymann et al. 2021). However, evidence is now accumulating about its potentially negative effects on humans and other vertebrates (Giaquinto et al. 2017; Gill et al. 2018; Van Bruggen et al. 2018). Given the significance of poultry products in human food consumption, it is important to pay a particularly close attention to how GLY affects poultry.

In laying hens (Fathi et al. 2019), Roundup^®^ administration (10 ppm GLY) was found to reduce hatchability, cause oxidative stress and lipid damage in exposed chickens. The level of GLY residue in the feed was shown to be negatively correlated with hatchability in broiler breeders (Foldager et al. 2021). Negative effects of a number of pesticides on the blood and immunity parameters of broilers have been identified including a decreased white blood cell count (WBC), lower numbers of T-lymphocytes, B-cells and nitro-tetrazolium-positive cells, and reduced spleen and thymus weight (Garg et al. 2004). In a previous study (Laptev et al. 2022), we showed that, even when feedstuffs for broilers were contaminated with GLY at an amount corresponding to 1 MRL, its negative effect on the expression of immunity genes in poultry was observed. It is therefore important to accurately establish the effects of different levels of GLY on poultry in order to possibly revise existing GLY-related regulations in the future animal production practice.

The aim of the current study was to conduct a comprehensive evaluation of the effects of different concentrations of chronic exposure to GLY on zootechnical characteristics and clinical, biochemical and immunological blood parameters, as well as post mortem weight and digestive organ length in Ross 308 broiler chickens. In this investigation, we established how broilers fed the GLY-containing diets responded in terms of changes in these economically important traits that could affect broiler production.

## Materials and methods

### Diets, animals, and experimental design

The experiments were conducted in a vivarium located in the village of Fedorovskoye, Tosnensky District, Leningrad Oblast in May–June 2022 using broiler chickens of a widely used Ross 308 cross (e.g., Bondarenko et al. 2013) that were reared for 35 days. Feeding and housing conditions met the requirements for broilers of this cross (Aviagen 2002, 2014; Egorov et al. 2013). The complete mixed fodder PK5-1G-1101 (CJSC Gatchinsky Feedmill, Leningrad Oblast, Russia) was used for feeding broilers aged from 1 to 4 weeks. From Day 28 to Day 35, the chicks were fed the complete mixed fodder PK-6-G-1102 (CJSC Gatchinsky Feedmill) (see details in Supplementary Table 1). In addition, the birds received an additional vitamin and mineral supplement (see details in Supplementary Table 2). With 65 birds each, four groups were formed. These included three experimental groups (II–IV) fed diets supplemented with GLY at doses of 10, 20, and 100 ppm that corresponded to 0.5, 1, and 5 MRL (SanPiN 2021), respectively, as well as an untreated control group (I). Regimes of watering, lighting and humidity were consistent with the Ross 308 broiler management guidelines (Aviagen 2002; Egorov et al. 2013). The experimental and control group chickens were kept in three tiered cages consisting of BB-1 blocks (NPO Stimul-INK, Pushkino, Moscow Oblast, Russia).

In the current experiment, GLY was used as a component of the Agrokiller preparation (CJSC Avgust, Moscow, Russia) that contained 500 g/l GLY acid (isopropylamine salt). For this purpose, the Agrokiller working solution was prepared and applied on mixed fodder by the spraying method. Mixing was carried out mechanically with observance of personnel safety requirements. After the application of GLY, its concentration in the feedstuffs was monitored by the enzyme-linked immunosorbent assay (ELISA; e.g., Tereshchenko and Ryabinin 2009). Due to the fast weight gain and increased feed intake in growing broilers, the dose of glyphosate was calculated in ppm (per 1 kg of compound feed). The average daily consumption of glyphosate per 1 broiler was 0.95 mg in Group II, 1.90 mg in Group III, and 9.51 mg in Group IV. The concentration of glyphosate in the feed was corrected by ELISA analysis to eliminate inevitable losses when an aerosol containing glyphosate was added to the feed. Importantly, the broiler diet contained almost no background traces of GLY, which indicated the purity of the experiment. For the ELISA analysis of GLY content in the feed and nutrient media, a Stat Fax 303 + strip immunoassay (Awareness Technology, Inc., Palm City, FL, USA) and a GLY ELISA Microtiter Plate test system (Eurofins Abraxis, Warminster, PA, USA) were used. The test is based on a direct competitive immunoassay reaction between GLY, which is present in the sample, and a GLY-labelled enzyme to bind rabbit antibodies to GLY and goat antibodies to rabbit immobilized in microtiter plates. After performing the immunoassay in the wells, the intensity of the color signal of the solution is inversely proportional to the concentration of GLY present in the samples. The content of glyphosate in feed in the experimental groups (II–IV) was 10.0 ± 0.6, 20.0 ± 1.8 and 100.0 ± 7.4 ppm, respectively.

### Zootechnical analyses

The zootechnical analyses were performed as recommended elsewhere (Egorov et al. 2013). Body weight gain (BWG) was analyzed on a weekly and individual basis as the body weight (BW) difference between the end and the beginning of a specific rearing period. Survival rate (SR) was defined as the ratio of the number of chickens that survived by the end of rearing to the initial number of chickens that was expressed as a percentage. Feed conversion rate (FCR) was determined as the ratio of total feed to total BWG. The coefficient of flock uniformity by BW (CV) was calculated as the number (expressed as a percentage) of birds weighed at 35 days of age that had a BW within ± 15% of average value. The European Productivity Index (EPI) was calculated using the following formula (Attia et al. 2014, 2020a, b):

$$ EPI=\frac{BW\times SR}{PP\times FCR},$$

where BW is body weight in kg, SR is survival rate in %, PP is production period in days, and FCR is feed conversion rate.

### Serum analyses

Laboratory examination of blood samples from chickens was carried out at 1, 7, 14 and 35 days of rearing at the Department of Biochemistry and Physiology, the Federal State Budgetary Educational Institution of Higher Education “St. Petersburg State Academy of Veterinary Medicine”. Conventional methods of laboratory analyses were used as follows. The number of blood erythrocytes was identified in a counting chamber with the Goryaev’s grid in accordance with the generally accepted method described elsewhere (Titz 1997). WBC was determined using a counter chamber with the Goryaev’s grid (Titz 1997). Blood leukograms were obtained by microscopy using May–Grünwald fixation solution and Romanowsky azure–eosin dye for staining blood smears (Titz 1997). Serum hemoglobin levels were estimated by the hemoglobin cyanide method (Titz 1997). Phagocytosis values were determined microscopically using a *Staphylococcus aureus* culture (strain 209) inactivated by heating and standardized using an optical turbidity standard (Menshikov 1997). Lysozyme activity was determined by the photoelectrocolometric method according to Dorofeichuk (as reviewed in Sadovnikov et al. 2009), with a change in the temperature mode of chicken blood serum lysis reaction using a culture of *M. lisodecticus*. Serum bactericidal activity (SBA) was determined as *E. coli* lysis percentage using the Michel Teffer method modified by Smirnova and Kuzmina (as reviewed in Sadovnikov et al. 2009). Determination of immunoglobulin classes was performed by the discrete precipitation method according to Kostina (1983). Phagocytic activity was expressed as a percentage of leucocytes involved in phagocytosis to the total number of neutrophil leucocytes counted (Karput 1993). Phagocytic index that describes the intensity of phagocytosis was calculated as the average number of phagocytosed bacteria in one leucocyte (Kolyakov 1990). Phagocytic number conformed to an average number of bacteria absorbed by one leucocyte (Kolyakov 1990). Concentrations of total protein, albumin, globulin, creatinine, bilirubin and uric acid as well activity of amylase, alkaline phosphatase, alanine aminotransferase and aspartate aminotransferase enzymes were found using the techniques described elsewhere (e.g., Nikolaienko et al. 2011) and the respective commercial kits (SPF Abris+, St. Petersburg, Russia).

The post mortem weight and length of broilers’ digestive organs were determined in 10 birds per group selected at random on Day 35. The birds were individually weighed and then killed by decapitation; after exsanguination, the thoracic cavity was opened with complete removal of internal organs. The length of the intestine from stomach to cloaca and the length of the glandular and muscular stomachs were measured. Liver weight, intestine weight, small intestine weight and stomach weight (without cuticle) were determined using a laboratory scale (Supplementary Fig. 1). A veterinary evaluation of the internal organs was carried out following the procedure describe elsewhere (Azimov 1971).

### Statistical analyses

Mathematical and statistical processing of the results was performed by multivariate analysis of variance (multi-factor ANOVA) in Microsoft Excel XP/2003 and RStudio (Version 1.1.453; RStudio Team 2018). The results are presented as mean (M) and standard errors of the mean (± SEM). Significance of differences was established by Student’s t-test, differences were considered statistically significant at *P* < 0.05. Mean values were compared using Tukey’s Significant Difference Test (HSD) and TukeyHSD function in R Stats Package (RDocumentation).

## Results

### Analyses of zootechnical characteristics

**Fig. 1:**
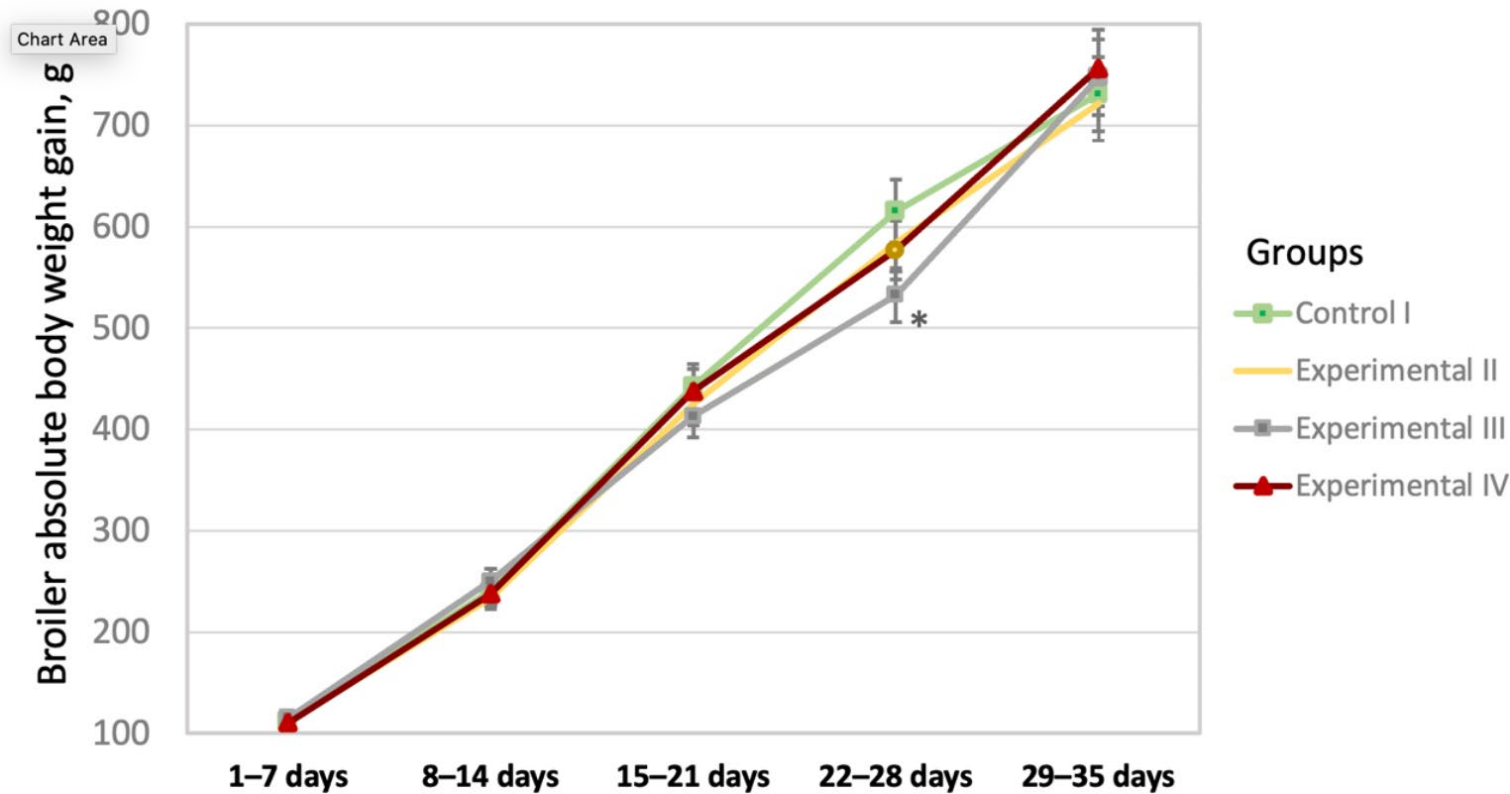
Changes in absolute body weight gain by weekly growing periods (1–7, 8–14, 15–21, 22–28, and 29–35 days of age) in response to GLY intake in the four groups (I–IV) of Ross 308 broiler chickens. *Significant difference between Groups III and I at *P* < 0.05. Results are presented as mean value (± SEM).

The results of changes in absolute BWG in Ross 308 broiler chickens by weekly rearing periods of the total experiment are shown in Fig. 1. At days 22–28 of growing, there was a decline in BWG by 83 g in Group III (20 ppm) as compared to Group I (*P* < 0.05), whereas Group IV (100 ppm) had no significant difference with Group I. At 29–35 days of age, the difference between the four groups was also insignificant (*P* > 0.05).

Zootechnical indicators such as SR, CV, BW, FCR and EPI at 35 days of age are presented in Table 1. SR seemed to be as high as 98.5% in Group I; however, no significant differences in SR, BW, FCR and EPI were established between the groups. There was a tendency for the lowest CV value in Group I (11.27%) as compared to the GLY-treated groups, with that in Group II being significantly greater by 1.94% (*P* < 0.05).

**Table 1:** Zootechnical characteristics of Ross 308 broiler chickens at 35 days of age in response to GLY intake

Response traits	Groups			
	I	II	III	IV
Survival rate, %	98.5	93.9	96.9	95.4
Body weight at 35 days of age, g*	2188.5 ± 41.1	2125.3 ± 43.8	2106.3 ± 39.6	2166.0 ± 39.6
Coefficient of flock uniformity by body weight, %	11.27	13.21**	12.33	12.00
Body weight of hens, g*	2053.7 ± 51.3	2021.9 ± 47.7	1974.8 ± 46.4	1994.9 ± 48.8
Body weight of cockerels, g*	2291.2 ± 50.2	2181.8 ± 48.8	2315.0 ± 46.4	2322.3 ± 51.6
Feed conversion rate	1.75	1.65	1.67	1.67
European Productivity Index	352	345	349	353

*Note*: *Presented as mean value (± SEM); **significant difference at *P* < 0.05 in comparison with Group I (as estimated by the Student’s *t*-test)

### Clinical and biochemical blood parameters

The results of the clinical and biochemical blood tests can be seen in Table 2 and Supplementary Table 3. We established that the number of erythrocytes in the blood of broilers in Group I at 35 days of rearing was higher than that on Day 7 (*P* < 0.05). This parameter in Groups II–IV on Day 7 tended to be greater than that in Group I, being further levelled by Day 35 of rearing, except Group IV where it was lower as compared to Group I (*P* < 0.05; Table 2). At the same time, the number of erythrocytes in the blood of 35-day-old birds in Groups II–IV did not change significantly as compared to 7-day-old broilers (*P* > 0.05). The hemoglobin concentration on Day 7 tended to be greater in GLY-fed broilers than in the untreated control. By 35 days of rearing, this parameter reached 101.7 ± 9.18 g/l in Group I and did not differ in Groups II and III, while being significantly lower in Group IV (79.0 ± 2.16; *P* < 0.05). In addition, there was an increased WBC in Group IV on Day 14 in comparison with Group I (4.0 ± 1.20 × 10^4^ vs. 2.1 ± 0.44 × 10^4^ μl^− 1^; *P* < 0.05; Table 2), being levelled by 35 days of age and not differing significantly in Groups II and III. The platelet count on Day 35 was significantly lower in Group IV as compared to Group I (*P* < 0.05), whereas it did not differ significantly in Groups II and III from that in Group I during the whole rearing period.

**Table 2:** Means (± SEM) of clinical and biochemical blood parameters and blood color index of Ross 308 broiler chickens in response to GLY intake (*n* = 3 per group)

Age, days	Groups			
	I	II	III	IV
Number of erythrocytes, × 10^6^ μl^− 1^ (norm range: 1.5–2.3 × 10^6^ μl^− 1^)				
1*	2.2 ± 0.2			
7	1.5 ± 0.3	1.9 ± 0.1	1.9 ± 0.1**	1.65 ± 0.2
14	1.9 ± 0.0	1.7 ± 0.2	1.8 ± 0.2	1.86 ± 0.1
35	2.1 ± 0.2	2.1 ± 0.1	1.9 ± 0.2	1.6 ± 0.1**
White blood cell count, × 10^4^ μl^− 1^ (norm range: 1.9–3.6 × 10^4^ μl^− 1^)				
1*	1.7 ± 0.2			
7	1.9 ± 0.2	2.1 ± 0.4	1.9 ± 0.3	2.3 ± 0.1
14	2.1 ± 0.4	2.5 ± 0.5	1.6 ± 0.2	4.0 ± 1.2**
35	2.0 ± 0.1	1.9 ± 0.2	2.3 ± 0.3	2.0 ± 0.4
Platelet count, × 10^4^ μl^− 1^ (norm range: 3.2–10.0 × 10^4^ μl^− 1^)				
1*	4.9 ± 2.1			
7	5.8 ± 1.9	4.9 ± 0.8	3.7 ± 0.8	5.9 ± 1.4
14	6.2 ± 1.1	3.9 ± 0.4**	4.0 ± 1.2	4.9 ± 1.6
35	4.2 ± 1.2	3.4 ± 1.2	4.2 ± 1.7	6.4 ± 0.2**
Hemoglobin concentration, g/l (norm range: 80–120 g/l)				
1*	92.3 ± 4.9			
7	73.7 ± 3.3	88.0 ± 4.9**	88.7 ± 1.9**	76.0 ± 2.8
14	84.0 ± 5.0	81.7 ± 7.9	88.7 ± 3.3	94.3 ± 12.8
35	101.7 ± 9.2	95.7 ± 6.6	95.3 ± 9.4	79.0 ± 2.2**
Blood color index (norm range: 2–4)				
1*	2.5 ± 0.2			
7	3.0 ± 0.5	2.9 ± 0.3	2.7 ± 0.2	2.8 ± 0.3
14	2.7 ± 0.2	2.9 ± 0.4	3.1 ± 0.4	3.1 ± 0.5
35	2.9 ± 0.1	2.8 ± 0.1	3.0 ± 0.1	2.9 ± 0.1

*Note*: *As determined before GLY administration; ** significant difference at *P* < 0.05 in comparison with Group I (as estimated by the Student’s *t*-test)

The results of biochemical analyses of broilers’ blood serum showed (Table 3) that the amylase content in the 35-day-old birds of Group IV was lower than that in Group I (*P* < 0.05). In Group III, this indicator was lower than in Group I in the first half of rearing (*P* < 0.05), being levelled later on. The ALP content changed somewhat inconsistently in Groups II, III and IV as compared to Group I. For example, the values of this indicator were in Groups II and IV on Day 7 lower and in Groups II and III at 14 days of age greater (by 14.6 and 7.6 IU, respectively) in comparison with Group I (*P* < 0.05). However, these differences turned to be insignificant at older ages, except the ALP amount in Group II at 35 days of age where it declined as compared to group I (*P* < 0.05). Interestingly, the changes in ALAT and ASAT values due to GLY intake followed similar patterns. Particularly, the values of these two parameters decreased at 14 days of age in Groups II, III, and IV in comparison with Group I (*P* < 0.05), being levelled later on. Blood uric acid content (Supplementary Table 5) was lower than normal in all samples (*P* < 0.05), except this parameter on Day 35 in Group III.

**Table 3:** Means (± SEM) of biochemical blood parameters in Ross 308 broiler chickens, including amylase, alkaline phosphatase (ALP), alanine aminotransferase (ALAT) and aspartate aminotransferase (ASAT) activities, in response to GLY intake (*n* = 3 per group)

Groups	Age, days	Amylase, g/hr/l	ALP, IU	ALAT, IU/l	ASAT IU/l
	1*	34.5 ± 1.1	138.0 ± 12.6	1.4 ± 0.1	19.5 ± 0.9
I	7	28.7 ± 0.9	115.9 ± 3.6	1.9 ± 0.2	36.7 ± 5.2
	14	21.8 ± 1.5	107.5 ± 2.5	2.8 ± 0.4	39.4 ± 3.2
	35	16.5 ± 0.7	85.5 ± 7.1	2.4 ± 0.3	23.1 ± 1.5
II	7	26.3 ± 2.0	97.5 ± 5.1**	2.1 ± 0.3	36.0 ± 3.1
	14	18.5 ± 0.8	122.1 ± 2.2**	1.8 ± 0.1**	28.8 ± 1.8**
	35	18.5 ± 0.8	73.3 ± 2.8**	1.6 ± 0.2**	23.5 ± 0.9
III	7	23.1 ± 1.2**	112.1 ± 2.5	1.5 ± 0.1	33.8 ± 3.9
	14	17.3 ± 1.1**	115.1 ± 2.4**	2.4 ± 0.1**	28.6 ± 2.2**
	35	15.91 ± 0.4	90.0 ± 10.8	2.55 ± 0.2	23.15 ± 1.5
IV	7	27.5 ± 0.7	97.9 ± 5.6**	2.1 ± 0.1	36.7 ± 3.2
	14	21.4 ± 0.9	101.0 ± 3.9	1.6 ± 0.1**	28.8 ± 1.8**
	35	13.5 ± 0.5**	85.5 ± 6.1	2.2 ± 0.4	24.2 ± 3.1
Norm range		15.0–30.0	**–**	1.2–6.8	22.0–50.0

*Note*: *As determined before GLY administration; ** significant difference at P < 0.05 in comparison with Group I (as estimated by the Student’s t-test)

### Immunological blood tests

**Fig. 2:**
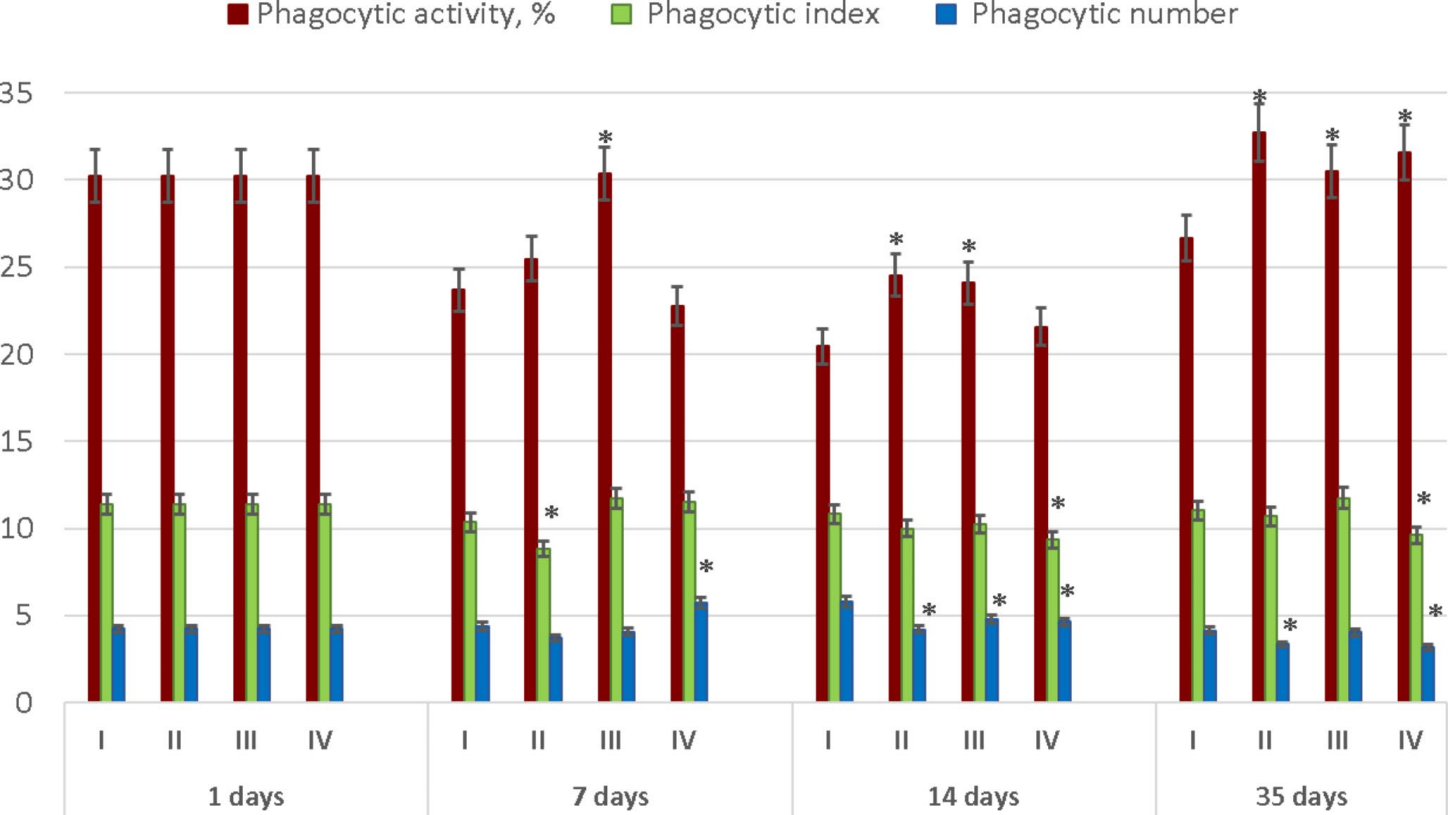
Blood phagocytic activity in response to GLY intake in the four Ross 308 broiler chicken groups (I–IV). *Significant difference in comparison with Group I (at *P* < 0.05). Results are presented as mean values (± SEM).

The levels of the phagocytic activity of broiler blood in response to GLY intake are shown in Fig. 2. There was a tendency toward increased values of phagocytic activity of leukocytes in GLY treatment variants as compared to Group I (*P* < 0.05) throughout the whole rearing period (Fig. 2). For example, in 35-day-old broilers, the phagocytic activity in Group I was 26.7 ± 1.22%, whereas in Groups II to IV it was greater (30.5 ± 5.87 to 32.7 ± 3.86%; *P* < 0.05). In addition, GLY administration at the highest concentration (Group IV) by 14 and 35 days of rearing resulted in lower phagocytic index values in comparison with Group I (*P* < 0.05; Fig. 2). By 14 and 35 days of the experiment, broilers’ phagocytic number declined in almost all experimental groups (excluding Group III on Day 35) as compared to Group I (*P* < 0.05).

As shown in Table 4, there were no differences in the values of lysozyme activity between the experimental and control groups. However, there was a 1.7- and 1.8-fold elevation in immunoglobulin IgA in the blood of 7-day-old chicks from Groups II and IV, respectively (*P* < 0.05). By Day 35, there was an increase in IgG in Groups III and IV in comparison with Group I (*P* < 0.05). At the same time, IgM content did not change significantly due to GLY treatment.

**Table 4:** Means (± SEM) of immunological blood parameters in Ross 308 broiler chickens, including lysozyme and serum bactericidal (SBA) activities and concentration of immunoglobulins, in response to GLY intake (*n* = 3 per group)

Groups	Age, days	Lysozyme activity, %	SBA, %	Immunoglobulin concentration, g/l		
				IgA	IgM	IgG
I	1*	15.0 ± 0.8	37.6 ± 4.7	0.4 ± 0.0	1.0 ± 0.1	3.0 ± 0.1
	7	12.7 ± 0.5	42.1 ± 2.7	0.3 ± 0.2	1.2 ± 0.2	3.0 ± 0.4
	14	12.0 ± 0.8	52.0 ± 3.0	1.3 ± 1.0	1.9 ± 0.2	3.6 ± 0.0
	35	12.0 ± 1.6	60.5 ± 1.6	1.3 ± 0.1	2.2 ± 0.1	3.6 ± 0.1
II	7	11.7 ± 1.2	42.4 ± 2.9	0.4 ± 0.1**	1.3 ± 0.0	3.6 ± 0.5
	14	11.3 ± 1.2	54.1 ± 3.2	1.6 ± 0.0	2.1 ± 0.1	3.0 ± 0.2
	35	10.0 ± 0.8	61.2 ± 2.1	1.2 ± 0.0	2.0 ± 0.2	3.8 ± 0.2
III	7	10.7 ± 0.5	40.1 ± 2.1	0.2 ± 0.1	1.2 ± 0.1	3.7 ± 0.6
	14	9.7 ± 1.2	52.2 ± 2.9	1.6 ± 0.1	1.7 ± 0.2	3.1 ± 0.3
	35	12.0 ± 0.7	61.0 ± 1.2	1.3 ± 0.1	2.1 ± 0.1	4.1 ± 0.1**
IV	7	11.7 ± 0.5	40.8 ± 1.8	0.5 ± 0.1**	1.2 ± 0.1	3.6 ± 0.4
	14	10.0 ± 1.6	50.8 ± 1.7	1.4 ± 0.1	1.8 ± 0.3	3.6 ± 0.1
	35	12.0 ± 1.4	58.4 ± 4.9	1.3 ± 0.1	2.1 ± 0.1	4.0 ± 0.2**

*Note*: *As determined before GLY administration; **significant difference at *P* < 0.05 in comparison with Group I (as estimated by the Student’s *t*-test)

### Post-mortem evaluation of the digestive system

According to the veterinary assessment at autopsy, the internal organs of the birds had no pathological changes. Changes in the post mortem weight and length of the digestive organs of broilers are shown in Table 5. There was a tendency in the reduced liver and intestinal weights and intestinal length in response to GLY administration. In particular, these characteristics were significantly lower by 5.7, 5.7 g and 21.4 cm respectively in Group III in comparison with Group I (Table 5; *P* < 0.05).

**Table 5:** Means (± SEM) of post mortem weight and length of digestive system organs in Ross 308 broiler chickens at 35 days of age in response to GLY intake (*n* = 10 per group)

Indicators	Groups			
	I	II	III	IV
Liver weight, g	43.2 ± 1.6	39.8 ± 2.0	37.5 ± 2.0*	44.1 ± 1.7
Bowel length, cm	224.3 ± 8.0	219.8 ± 12.0	202.9 ± 6.9*	216.2 ± 6.8
Intestinal weight, g	62.5 ± 1.7	59.7 ± 1.5	56.8 ± 1.6*	61.4 ± 3.6
Small intestine weight, g	48.6 ± 1.e	47.1 ± 1.3	44.6 ± 5.1	48.5 ± 2.8
Large intestine weight, g	13.9 ± 1.w	12.6 ± 0.6	12.2 ± 1.0	13.0 ± 1.2
Stomach weight, g	27.7 ± 0.9	26.2 ± 1.1	26.3 ± 1.0	26.4 ± 1.3
Stomach length, cm	9.4 ± 0.2	9.5 ± 0.9	9.5 ± 0.2	9.6 ± 0.2

Note: *Significant difference (at *P* < 0.05) when comparing Groups II–IV to the control (I) as estimated by the Student’s *t*-test

## Discussion

Previously, GLY intake-related effects were examined at various concentrations of this nonselective herbicide in meat-type poultry diets. For example, Kubena et al. (1981) did not observe significant differences in performance and selected carcass traits in broilers at 60.8 and 608 ppm from 1 day to 21 days of age, while the highest dose (6080 ppm) dramatically affected BW and also elevated the calcium and magnesium concentration in the tibiotarsus bones. In this study, we carried out, for the first time, a comprehensive evaluation of the GLY effects at 10, 20 and 100 ppm on zootechnical characteristics and clinical, biochemical and immunological blood parameters, as well as post mortem weight and length parameters of the digestive organs in response to its chronic exposure among Ross 308 broiler chickens. We identified that at 22–28 days of rearing, the GLY dose of 20 ppm significantly led to a lower BWG, whereas no similar significant effect was found at other doses and ages. The absence of negative effect on bird BWG at a higher dosage (100 ppm) and its presence at 20 ppm can be associated, to our mind, with concomitant changes in physiological parameters of broilers. We hypothesize that birds treated with GLY at the concentration of 100 ppm may have altered the intestinal architecture by reducing villous height, which may have adversely affected the intestinal absorption ability. This could contribute to a decreased amount of GLY absorbed and consequently a decline in the GLY content in the body. Intake of a lower toxin dosage (20 ppm) may not have had the same effect on the villous structure, resulting in a greater absorption of this xenobiotic in the intestine and, consequently, a reduced productivity. At 29–35 days of age, this difference in absolute BWG between Groups I and III disappeared, possibly due to a transition to a different, more energy-rich diet (with metabolic energy of 315 kcal/100 g vs. 295 kcal/100 g). In addition, the chicks at 35 days of rearing are more physiologically developed and have more advanced enzyme, digestive and immune systems, which may allow them to compensate for the lag in growth caused by the introduction of GLY into the diet.

We also found that the presence of GLY in the poultry feedstuffs could be a stressor that tended to negatively affect the flock CV (Table 1); however, no dose increase-dependent effect on this parameter was observed. CV is an important indicator in the rearing of commercial poultry, especially high-yielding breeds (Kochish et al. 2007). Birds in a flock that is not uniform by BW are expected to show different responses to the rearing conditions provided by the conforming veterinary, zootechnical and technological (automated slaughtering and product processing) measures. This may lead to increased feed and energy costs, higher mortality and reduced productivity (Nkrumah et al. 2006; Bondarenko and Khvostik 2020). The EPI parameter (Attia et al. 2014, 2020a, b), which reflects production efficiency in poultry meat production by combining such indicators as BW, SR and FCR (Jackson et al. 1982; Patreva et al. 2010; Lilly et al. 2011). However, we did not identify any significant differences in the EPI values between Groups I to IV (Table 1). The negative effects of GLY intake on zootechnical performance may be due to the detrimental effects of this toxicant on both bird body (Gill et al. 2018; Bali et al. 2019) and its microbiome (Motta et al. 2018; Mesnage et al. 2021), although we were unable to prove this definitely here for all the studied zootechnical characteristics, suggesting a need in a more detailed investigation in the future.

The number of erythrocytes in the blood of broilers in Group I was greater on Day 35 than that on Day 7 (*P* < 0.05; Table 2). When GLY was fed at different dosages, the number of erythrocytes in the blood of 35-day-old broilers did not increase as compared to 7-day-old broilers. Normally, there is an elevation in erythrocyte count from an average of 1.6 to 3.4 × 10^6^ μl^− 1^ from 1 to 25 days of rearing (Kochish et al. 2007). A lowered erythrocyte count in the blood of birds under the influence of aflatoxin has also been shown previously (Dönmez et al. 2012). In the present investigation, we observed this significant adverse effect in 35-day-old broilers treated with the GLY supplementation at the highest dose (i.e., 100 ppm).

By Day 35 of rearing, GLY administration at the dose of 100 ppm also resulted in a significant decline in hemoglobin (*P* < 0.05; Table 2). The observed decrease in erythrocytes and hemoglobin due to GLY intake may be due to many factors, such as a drop in the general ability to bind iron (Harvey et al. 1991), defects in hematopoietic cells (van Vleet and Ferrans 1992), and inhibition of protein synthesis (Kaneko 1989). This is confirmed by the reduced level of α-globulin (5.0 ± 0.61 vs. 6.3 ± 0.52 g/l) in our experiment in Group III in comparison with Group I (Supplementary Table 4). A reduced α-globulin fraction can be observed in diabetes, pancreatitis and toxic hepatitis (Yarets 2015). Previously, Abdel-Wahhab et al. (2002) reported that aflatoxicosis also reduced hemoglobin and total erythrocyte counts in broilers. Interestingly, they demonstrated that changes in these parameters resulted in the normocytic normochromic anemia in birds.

On Day 14, there was an elevation in WBC in Group IV as compared to Group I (Table 2). Such an observation suggests that GLY-containing feedstuffs may have initiated an inflammatory response in birds. This is a highly negative fact because, according to Humphrey et al. (2014), the overproduction of modern broiler breeds, along with the use of high-energy diets oversaturated in fatty acids and carbohydrates, also has a negative effect on immune regulation in the gut. As a result, chronic systemic inflammation and increased metabolic disturbances occur. Activation of pro-inflammatory genes in birds influenced by the pesticide could be a mechanism for such negative effects. For example, we recently demonstrated that experimental T-2 toxicosis in broilers activates the expression of proinflammatory cytokine *IL6* (interleukin 6; up to 41.7-fold) and *PTGS2* (cyclooxygenase-2) genes involved in inflammation regulation in the pancreas (Yildirim et al. 2021). In an earlier study, Wang et al. (2018) observed increased expression of inflammation-related genes such as *NFKB1* (*NF-κB*), *NOS2* (*iNOS*), *COX2* (*COX-2*), *PTGES* (*PTGE*), *IL6* (*IL-6*), *LITAF* (*TNF-α*) and *IL-4* (*IL-4*) in chick spleen in response to selenium intake in concentrations higher than normal.

The results of biochemical analysis of broilers’ serum (Table 3) might suggest a slight toxic effect of GLY on broilers, as a decreased serum amylase level have previously linked to the negative effects of various toxicants such as ethanol (Maruyama et al. 2003) and aflatoxin (Ditta et al. 2019). The reduced uric acid content in the blood of broilers (Supplementary Table 5) was probably due to the low purine content in the feed.

We observed a tendency toward a higher phagocytic activity of leucocytes in some GLY treatment variants in comparison with Group I during the whole period of broiler rearing (Fig. 2). These results correlated with the reported here elevation in WBC by Day 14 in Group IV as compared to Group I (Table 2). The phagocytic activity of leucocytes is known to be an indicator that can grow in response to various stressors (Alhussien and Dang 2020). For example, temperature deviations within ± 10 °C from normative values during the maintenance of young chickens lead to the development of temperature stress and stimulation of phagocytic activity of blood leukocytes by 5–15% in comparison with the initial values (Tsarev 2018). In addition, GLY administration in some cases resulted in a lower phagocytic index and phagocytic count as compared to Group I (Fig. 2). We suggest that impaired immune responses were expressed as a decline in phagocytic index and phagocytic number in birds following administration of GLY in the feedstuffs. This can lead to increased frequency and severity of infectious diseases and opportunistic infections in livestock exposed to this toxicant.

Serum immunoglobulins A, G and M (IgA, IgG and IgM) are part of the adaptive immune system (Kislenko 2007). In the presence of GLY in the feed, we observed a rise in IgA content in the blood of 7-day-old chicks from Groups II and IV in comparison with Group I (Table 4). By Day 35, there was an increase in IgG in Groups III and IV as compared to Group I. At the same time, IgM content did not change due to GLY intake. Interestingly, IgM provides a rapid immune response and participates in tissue homeostasis, whereas IgG and IgA are long-term high-affinity antibodies, the latter mainly providing mucosal immunity (Hoffman et al. 2016). Therefore, the growth in IgA concentration on Day 7 may be related mainly to the immune response of the mucous membrane and the impaired intestinal barrier function of 1-week-old chickens because of GLY administration. Organisms in the early stages of development are generally more susceptible to external stress than adults (Jankord et al. 2011). In the case of environmental toxins, we hypothesize that this may be due to an underdeveloped detoxification metabolism in the young. The development of the digestive system and toxin defense mechanisms in chickens with age may have resulted in greater resistance to the GLY effects. Remarkably, elevated immunoglobulin levels may indicate the presence of inflammatory processes (Yang et al. 2018; Atkin et al. 2018). Higher immunoglobulin levels have been reported in the presence of psychological stress (Sorrells and Sapolsky 2007). Elevated serum IgA levels have been reported in the presence of ethanol (Purohit et al. 2008).

When administering GLY, liver and intestinal weights and intestinal length tended to be reduced in Group II–IV in comparison with Group I (Table 5). It is likely that the lowering of these indicators was associated with a tendency of the decreased absolute BWG of broilers during 22–28 days of rearing since underdevelopment of the intestine can lead to a reduction in enzymatic and absorptive activity. Deterioration of digestive structures can have a negative effect on the formation of gut-associated lymphoid tissue component of the lymphoid tissue related to mucosal-associated lymphoid tissue (MALT). MALT acts as the first line of defense against mucosal pathogens (Friedman et al. 2003). Thus, our data on the effects of GLY in broilers are consistent with current biological understanding of the digestive and immune systems. In line with our observations, negative effects of glyphosate, including hepatotoxicity, reproductive toxicity, and neurotoxicity, were shown in a few other experimental models (Pu et al. 2020; Fu et al. 2021; Lian et al. 2023).

In conclusion, human and environmental safety data of the nonselective GLY herbicides (Roundup^®^ and other brands) as reviewed and approved by global regulatory agencies suggest their safe application to control weeds with crops intended for animal feed production (Vicini et al. 2019, 2021; Heymann et al. 2021). There is however a growing concern regarding possible adverse effects. We have shown here that GLY intake with the feed could be a stressor that can have multiple effects on some economically important characteristics and blood parameters in broilers. The level of these effects varies at different stages of ontogenesis. GLY could be a stimulus that activates an increased count and phagocytic activity of white blood cells. Overall, we can suggest that excessive immune responses to antigenic stimulation of the gastrointestinal tract can be energetically costly, have a negative effect on broiler rearing efficiency and divert nutrients from building meat mass in the body. Disturbances in the immune system of poultry can have far-reaching negative consequences as commercial flocks are raised under intensive use conditions. They are vulnerable to the rapid spread of infectious agents, which can potentially cause large disease outbreaks. In this study, changes in blood parameters due to GLY intake have been associated with possible negative effects on zootechnical characteristics such as BWG and CV as well as adverse effects on digestive tract development.

On the other hand, the use of GLY at doses of 10, 20 and 100 ppm did not show an obvious, clearcut dose-dependent harmful effect on zootechnical characteristics and blood parameters of broilers. However, the present study suggested that exposure to the GLY-contaminated feedstuffs, even at legally permitted levels (0.5 and 1 MRL), could affect the physiological condition and performance of broilers.

Our findings indicate that GLY-induced chronic toxicosis can be diagnosed further by determining clinical, biochemical and immunological markers in serum (Katerinich et al. 2014). However, these parameters require further investigation and clarification. It is of interest to assess the mechanisms of the negative effects of GLY on poultry. These effects could be, for example, mediated by changes in the composition of the symbiotic gut microbiome and by induction or suppression of host gene expression (Laptev et al. 2021). Based on previous studies to examine the action mechanisms of other xenobiotics (Briggs and Briggs 2014), it can be suggested that glyphosate, like other chemical pollutants, can have both direct and indirect effects at several levels of body organization. However, further in-depth studies would be needed. Further research is also desirable to find agents with the ability to neutralize GLY residues in the feed or biodegrade them in the intestine of birds.

### Electronic supplementary material

Below is the link to the electronic supplementary material.

Supplementary Material 1

## Data Availability

All datasets used and/or analyzed during this study are included in this article and are available from the corresponding author on reasonable request.
