# Supplementary material for: Investigating adverse effects of chronic dietary exposure to herbicide glyphosate on zootechnical characteristics and clinical, biochemical and immunological blood parameters in broiler chickens
Source: Vet Res Commun. 2023 Aug 18;48(1):153–64. doi: 10.1007/s11259-023-10195-x (PMC10810961; doi:10.1007/s11259-023-10195-x)
Supplement: Supplementary file 1 — Supplementary Material 1 [file 11259_2023_10195_MOESM1_ESM.docx]

**Supplementary materials**

**Supplementary Table 1.** Composition and nutritional value of the broiler feed used

| **Components** | **Age of birds, days** | |
| --- | --- | --- |
|  | **1–27** | **28–35** |
| Wheat, % | 44.97 | 31.49 |
| Maize, % | 20.00 | 33.00 |
| Soya meal, % | 18.34 | 8.60 |
| Sunflower meal, % | 6.00 | 5.00 |
| Sunflower seed cake, % | 0 | 6.50 |
| Protein concentrate, % | 3.60 | 5.50 |
| Fish meal, % | 2.86 | 0 |
| Fodder yeast, % | 2.00 | 4.00 |
| L-lysine monohydrochloride, % | 0.49 | 0.52 |
| DL-methionine, % | 0.28 | 0.30 |
| L-threonine, % | 0.11 | 0.11 |
| Limestone meal, % | 0.03 | 0.40 |
| Monocalcium phosphate, % | 0 | 0.25 |
| Defluorinated phosphate | 0.16 | 0 |
| Table salt, % | 0.08 | 0.14 |
| Sodium sulphate, % | 0.08 | 0.09 |
| Premix P5 start U, % | 1 | 1 |
| 100 g of mixed fodder contains: | | |
| Exchangeable energy, Kcal/100 g | 295 | 315 |
| Humidity, % | 11.82 | 10.45 |
| Crude protein, % | 22.00 | 19.01 |
| Raw fat, % | 2.51 | 6.00 |
| Crude fibre, % | 3.80 | 4.26 |
| Ash in HCL, % | 0.35 | 0.89 |
| Linoleic acid, % | 1.12 | 3.05 |
| Lysine, % | 1.25 | 1.10 |
| Methionine, % | 0.60 | 0.58 |
| Methionine + cystine, % | 0.91 | 0.86 |
| Threonine, % | 0.82 | 0.73 |
| Tryptophan | 0.22 | 0.20 |
| Lysine YP, % | 0.55 | 0.98 |
| Methionine YP, % | 0.70 | 0.54 |
| Methionine + cystine YP, % | 0.77 | 0.76 |
| Threonine YP, % | 0.70 | 0.62 |
| Calcium | 0.90 | 0.82 |
| Phosphorus | 0.58 | 0.51 |
| Phosphorus | 0.44 | 0.39 |
| Potassium | 0.74 | 0.47 |
| Chlorine | 0.21 | 0.22 |
| Sodium | 0.16 | 0.16 |
| Sodium chloride | 0.35 | 0.40 |

**Supplementary Table 2.** Vitamin and mineral premix composition, per 1 kg of feed

| **Components** | **Rearing age, days** | |
| --- | --- | --- |
|  | **1–27** | **28–35** |
| Vitamin A, 10^4^ IU/kg ^-1^ | 1.4 | 1.1 |
| Vitamin D3, 10^3^ IU/kg ^-1^ | 5.00 | 5.00 |
| Vitamin E, mg/kg | 80.00 | 50.00 |
| Vitamin K3, mg | 4.00 | 3.00 |
| Vitamin B1, mg | 4.00 | 2.00 |
| Vitamin B2, mg | 9.00 | 8.00 |
| Vitamin B3, mg | 15.00 | 12.00 |
| Vitamin B4, mg | 400.00 | 350.00 |
| Vitamin B5 (niacin) RP, mg | 60.00 | 50.00 |
| Vitamin B6, mg | 4.00 | 3.00 |
| Vitamin Вс /Folium | 2.00 | 1.50 |
| Vitamin B12, mg | 0.020 | 0.020 |
| Vitamin H /biotin/, mg | 0.200 | 0.180 |
| Iron, mg | 40.00 | 40.00 |
| Manganese, mg | 100.00 | 100.00 |
| Zinc, mg | 100.00 | 100.00 |
| Copper, mg | 15.00 | 15.00 |
| Iodine, mg | 1.00 | 1.00 |
| Selenium, mg | 0.30 | 0.30 |
| Endox, mg | 124.00 | 125.00 |
| Rovabio Excel AP, mg | 50.00 | 50.00 |
| Chostazim P10000, mg | 50.00 | 50.00 |

**Supplementary Table 3.** Leukograms (%) from the blood of Ross 308 cross broiler chickens in response to glyphosate intake (M ± m, n = 3; vivarium, Fedorovskoye, Tosnensky District, Leningrad Oblast, 2022)

| Groups | Age of birds, days | Basophils | Eosinophils | Pseudo-eosinophils | | Lymphocytes | Monocytes |
| --- | --- | --- | --- | --- | --- | --- | --- |
|  |  |  |  | Bacillary | Segmentonuclear |  |  |
| Before feeding glyphosate | 1 | 1.0±0.00 | 2.3±0.47 | 27.1±4.83 | 0.3±0.47 | 64.1±10.19 | 1.3±0.47 |
| I | 7 | 1.3±0.47 | 1.7±0.94 | 22.0±7.12 | 0 | 73.0±6.48 | 1.7±0.47 |
|  | 14 | 0.7±0.47 | 1.3±0.47 | 22.3±1.25 | 0 | 73.3±2.36 | 2.3±1.25 |
|  | 35 | 0.7±0.47 | 2.7±1.70 | 27.3±1.89 | 0 | 68.3±1.89 | 1.0±0.82 |
| II | 7 | 1.3±0.94 | 2.0±0.82 | 27.3±1.25 | 0.33±0.47 | 68.0±1.41 | 1.3±0.47 |
|  | 14 | 0.7±0.47 | 1.3±0.47 | 30.3±2.87 | 0.33±0.47 | 67.0±2.45 | 0.3±0.47 |
|  | 35 | 1.7±0.94 | 0.7±0.47 | 29.0±1.41 | 0 | 68.0±2.83 | 0.7±0.94 |
| III | 7 | 1.0±0.00 | 1.0±0.00 | 25.7±6.18 | 0.33±0.47 | 70.3±7.59 | 1.7±1.25 |
|  | 14 | 1.7±0.94 | 1.7±1.25 | 31.7±7.32 | 0 | 63.7±9.10 | 1.7±0.47 |
|  | 35 | 2.0±0.82 | 1.3±0.47 | 30.3±1.89 | 0.3±0.47 | 64.7±1.25 | 1.3±0.47 |
| IV | 7 | 0.3±0.47 | 0.7±0.47 | 28.7±2.05 | 0 | 69.3±2.05 | 1.0±0.00 |
|  | 14 | 0.7±0.47 | 1.0±0.00 | 32.7±1.25 | 0.3±0.47 | 64.7±1.25 | 0.7±0.94 |
|  | 35 | 1.3±1.25 | 0.7±0.47 | 29.3±2.87 | 0 | 67.3±3.30 | 1.0±0.00 |

Note. Groups: I, Control; II, III, IV, Experimental

**Supplementary Table 4.** Results of total protein and proteinogram of blood of Ross 308 cross broiler chickens in response to glyphosate feeding (M ± m, n = 3; vivarium, Fedorovskoye, Tosnensky District, Leningrad Oblast, 2022)

| Groups | Age of birds, days | Total protein, g/l | Albumins, | | Globulins | | | | | | Albumin– globulin ratio |
| --- | --- | --- | --- | --- | --- | --- | --- | --- | --- | --- | --- |
|  |  |  | % | g/l | α, % | α, g/l | β, % | β, g/l | γ, % | γ, g/l |  |
|  | 1 | 20.2±0.61 | 50.9±1.26 | 10.3±0.51 | 12.3±1.05 | 2.46±0.19 | 16.8±0.43 | 3.4±0.01 | 20±0.63 | 4.0±0.10 | 1.1 ±0.05 |
| I | 7 | 24.4±1.69 | 52.2±1.27 | 12.8±1.06 | 13.1±1.35 | 3.2±0.46 | 13.8±0.96 | 3.3±0.03 | 20.9±1.78 | 5.1±0.37 | 1.1±0.05 |
|  | 14 | 31.3±0.98 | 54.3±0.85 | 17.0±0.61 | 14.8±2.18 | 4.6±0.57 | 9.1±2.62 | 2.9±0.91 | 21.8±0.92 | 6.8±0.19 | 1.2±0.04 |
|  | 35 | 40.5±1.06 | 58.0±1.64 | 23.2±1.24 | 15.8±1.78 | 6.3±0.52 | 8.4±0.22 | 3.4±0.18 | 17.7±0.19 | 7.1±0.19 | 1.4±0.09 |
| II | 7 | 26.2±1.68 | 52.2±2.33 | 13.7±1.05 | 16.0±1.91 | 4.2±0.73 | 12.7±3.26 | 3.3±0.69 | 19.147±1 | 5.0±0.38 | 1.1±0.10 |
|  | 14 | 31.1±1.65 | 53.2±1.83 | 16.6±1.03 | 16.2±1.55 | 5.0±0.48 | 9.3±0.88 | 2.9±0.39 | 21.4±0.63 | 6.7±0.12 | 1.1±0.08 |
|  | 35 | 43.4±0.78 | 52.8±3.97 | 22.9±1.37 | 15.4±0.71 | 6.7±0.39 | 15.8±4.65 | 6.9±2.08 | 16.0±0.66 | 7.0±0.26 | 1.1±0.19 |
| III | 7 | 24.5±1.63 | 52.3±0.85 | 12.8±0.95 | 16.4±0.81 | 4.0±0.21 | 10.3±2.07 | 2.5±0.37 | 21.0±1.42 | 5.2±0.62 | 1.1±0.04 |
|  | 14 | 33.2±0.4 | 55.0±2.37 | 18.2±0.73 | 16.4±0.77 | 5.4±0.29 | 9.2±0.97 | 3.1±0.35 | 19.3±0.95 | 6.4±0.32 | 1.2±0.11 |
|  | 35 | 39.4±0.57 | 59.1±1.34 | 23.3±0.2 | 12.6±1.5 | 5.0±0.61 | 9.4±2.51 | 3.7±1.01 | 19.0±0.48 | 7.5±0.11 | 1.5±0.48 |
| IV | 7 | 25.0±3.57 | 53.4±2.22 | 13.4±1.87 | 15.5±1.15 | 4.0±0.72 | 10.2±2.7* | 2.6±0.79 | 21.0±1.66 | 5.2±0.27 | 1.2±1.10 |
|  | 14 | 33.8±1.1 | 55.9±1.6 | 18.9±0.34 | 13.4±1.95 | 4.6±0.80 | 10.5±0.83 | 3.6±0.28 | 20.1±0.69 | 6.8±0.18 | 1.3±0.08 |
|  | 35 | 41.8±1.1 | 56.4±3.13 | 23.6±1.24 | 14.3±0.72 | 6.0±0.25 | 11.5±4.48 | 4.8±1.92 | 17.9±0.71 | 7.5±0.19 | 1.3±0.16 |

Note. Groups: I, Control; II, III, IV, Experimental

**Supplementary Table 5.** Results of bilirubin, uric acid and creatinine blood concentrations in broiler chickens Ross 308 in response to glyphosate feeding (M ± m, n = 3; vivarium, Fedorovskoye, Tosnensky District, Leningrad Oblast, 2022)

| Groups | Age of birds, days | Bilirubin, mmol/l | | Uric acid, μmol/l | | Creatinine, μmol/l | |
| --- | --- | --- | --- | --- | --- | --- | --- |
|  |  | Actual value | Norm | Actual value | Norm | Actual value | Norm |
|  | 1 | 1.6±0.19 | 0.94–7.5 | 322.1±26.53 | 360–560 | 20.7±1.07 | 20–87 |
| I | 7 | 2.7±0.17 | 0.94–7.5 | 325.5±35.88 | 360–560 | 31.1±1.07 | 20–87 |
|  | 14 | 1.8±0.15 |  | 293.8±20.8 |  | 36.9±1.06 |  |
|  | 35 | 3.4±0.14 |  | 338.8±15.82 |  | 44.9±1.07 |  |
| II | 7 | 1.7±0.25 | 0.94–7.5 | 289.8±15.11 | 360–560 | 34.3±0.84 | 20–87 |
|  | 14 | 1.7±0.24 |  | 277.2±10.89 |  | 31.6±1.95 |  |
|  | 35 | 3.1±0.24 |  | 294.9±19.7 |  | 46.4±0.83 |  |
| III | 7 | 2.4±0.12 | 0.94–7.5 | 353.1±9.2 | 360–560 | 30.7±0.79 | 20–87 |
|  | 14 | 2.2±0.13 |  | 320.2±4 |  | 36.3±1.94 |  |
|  | 35 | 3.7±0.37 |  | 388.7±11.33 |  | 48.1±1.36 |  |
| IV | 7 | 2.0±0.09 | 0.94–7.5 | 274.9±9.16 | 360–560 | 32.8±1.16 | 20–87 |
|  | 14 | 2.2±0.18 |  | 275.3±6.76 |  | 34.6±1.36 |  |
|  | 35 | 3.1±0.07 |  | 307.2±17.6 |  | 49.0±1.71 |  |

Note. Groups: I, Control; II, III, IV, Experimental


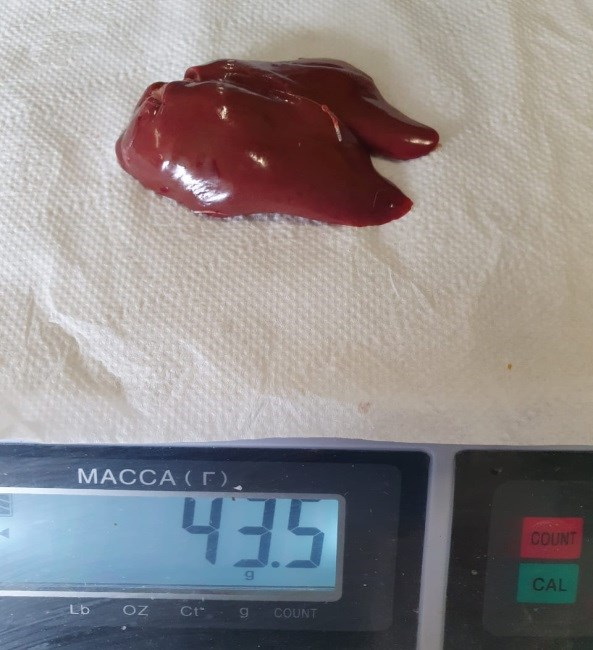


**Supplementary Figure 1.** Weighing a liver sample using a laboratory scale
